# Editorial: Autophagy in solid tumors and inflammation: mechanisms and therapies

**DOI:** 10.3389/fonc.2026.2016779

**Published:** 2026-10-20

**Authors:** Zhenyi An, Safa Larafa, Silvia Vega Rubin de Celis

**Affiliations:** 1 Broad Institute of Massachusetts Institute of Technology (MIT) and Harvard, Cambridge, MA, United States; 2 Koch Institute for Interactive Cancer Research, MIT, Cambridge, MA, United States; 3 Institute for Cell Biology (Cancer Research), University Hospital Essen, Essen, Germany

**Keywords:** autophagy, chaperon mediated autophagy (CMA), inflammation, therapies, tumor

Autophagy is a fundamental cellular quality-control and recycling process that maintains metabolic and proteostatic homeostasis under physiological and stressful conditions. In cancer, however, its functions are highly context-dependent. Depending on tumor type, genetic background, disease stage, and therapeutic pressure, autophagy may suppress early tumorigenesis while promoting survival, adaptation, progression, and treatment resistance in established tumors. Increasing evidence further indicates that selective forms of autophagy, including chaperone-mediated autophagy (CMA) and mitophagy, regulate processes extending beyond intracellular degradation, including metabolism, inflammation, immune regulation, cellular plasticity, and therapeutic resistance. Defining these diverse functions is therefore essential for determining when and how autophagy can be therapeutically exploited.

Therapeutic targeting of autophagy remains challenging because the pathway is closely integrated with major oncogenic signaling networks and cellular stress responses. Autophagy interacts with PI3K/AKT/mTOR and AMPK signaling, apoptosis, and mitochondrial quality-control mechanisms, enabling tumor cells to adapt to hypoxia, nutrient deprivation, targeted therapy, chemotherapy, and other forms of stress. Moreover, differences in autophagy dependence across tumor types and disease stages raise important questions regarding patient selection, treatment timing, and biomarker development. This collection, *Autophagy in Solid Tumors and Inflammation: Methods and Therapies*, brings together six articles, comprising five reviews and one original research study, that examine these challenges across diverse solid tumors.

The review by Tiwari et al. provides a comprehensive overview of CMA and its contribution to cancer biology. CMA selectively recognizes soluble proteins containing KFERQ-like motifs and delivers them to lysosomes for degradation. Although traditionally considered a mechanism of protein quality control and stress adaptation, CMA has broader effects on tumor biology. Dysregulated CMA can influence sustained proliferative signaling, resistance to cell death, metabolic reprogramming, invasion and metastasis, immune evasion, genome instability, and tumor-promoting inflammation. The review also highlights roles for CMA in cellular plasticity and cancer stem cell maintenance, processes strongly associated with tumor progression and therapeutic resistance. Collectively, these findings demonstrate that selective autophagy can regulate cancer through targeted degradation of key regulatory proteins. The authors emphasize, however, that the context-dependent nature of CMA necessitates the identification of appropriate biomarkers and therapeutic windows before CMA-targeted strategies can be translated clinically.

Fang et al. examine the dual and stage-dependent functions of autophagy in thyroid cancer, encompassing papillary, follicular, medullary, and anaplastic thyroid carcinomas. The authors propose that the transition from tumor-suppressive to tumor-promoting autophagy is associated with disease progression and represents a largely unidirectional shift toward an adaptive, tumor-supportive state rather than a simple reversible equilibrium. Major genetic drivers of thyroid cancer, including BRAF V600E, RAS, and RET, regulate autophagy through distinct mechanisms, underscoring the importance of tumor genotype in determining autophagy dependence. The authors further propose that autophagy primarily functions as an adaptive resistance mechanism rather than a primary oncogenic driver, consistent with the absence of established autophagy gene mutations as major drivers of thyroid cancer. Accordingly, autophagy inhibitors may be more effective in combination with treatments that impose metabolic or cellular stress than as monotherapies. The review supports biomarker-enriched, hypothesis-driven clinical trials, particularly in anaplastic thyroid carcinoma, and highlights combinations such as hydroxychloroquine with tyrosine kinase inhibitors.

The mini-review by Yan et al. focuses on mitophagy and its contribution to treatment resistance in solid tumors. Because mitochondria regulate metabolism, redox balance, energy production, and apoptosis, mitochondrial quality control is critical for malignant cells exposed to therapeutic stress. By selectively eliminating damaged mitochondria, mitophagy can preserve mitochondrial fitness and promote tumor-cell survival under unfavorable conditions. The review summarizes evidence linking mitophagy to tumor survival, proliferation, and resistance to anticancer treatments, reinforcing the concept that autophagy enables tumor cells to adapt to therapeutic stress. Defining the molecular mechanisms that regulate mitophagy may therefore reveal vulnerabilities that can be targeted in combination with established anticancer therapies.

Qin et al. review the interplay between autophagy and apoptosis in non-small cell lung cancer (NSCLC), with particular emphasis on natural products as potential therapeutic agents. Autophagy and apoptosis are distinct but interconnected processes whose balance can determine whether stressed tumor cells survive or undergo cell death. The review summarizes the molecular mechanisms and major points of crosstalk between these pathways and examines their relevance to NSCLC. Natural products are of particular interest because of their diverse biological activities and capacity to influence multiple molecular targets. The authors summarize natural compounds investigated for their effects on autophagy and apoptosis and discuss four representative agents in greater detail. Their analysis highlights the therapeutic potential of simultaneously targeting interconnected survival and cell-death pathways.

Xia et al. address autophagy-mediated resistance to PI3K/AKT/mTOR pathway inhibition in glioblastoma (GBM), an aggressive and highly treatment-resistant tumor frequently characterized by activation of this signaling axis. Although PI3K/AKT/mTOR inhibition represents an attractive therapeutic strategy, treatment-induced autophagy may provide a cytoprotective response that enables tumor cells to adapt. Pharmacological inhibition of this pathway can therefore activate autophagy, potentially contributing to both therapeutic responses and subsequent resistance. The review examines the rationale for combining autophagy inhibitors with PI3K/AKT/mTOR inhibitors to overcome this adaptive response. This work exemplifies a central theme of the collection: the therapeutic value of autophagy inhibition depends on identifying contexts in which autophagy becomes a critical survival mechanism following targeted treatment.

Finally, the original research article by Alanazi et al. investigates the relationship between autophagy-related gene expression, Epstein–Barr virus (EBV) infection, and clinical outcomes in nasopharyngeal carcinoma (NPC). The study evaluated nine autophagy-related genes at the mRNA level by quantitative PCR and assessed protein expression by immunohistochemistry in 35 NPC specimens and five non-cancerous nasopharyngeal mucosa controls. Several genes, including ATG3, ATG4D, ATG4C, ATG4A, ATG2B, and ATG5, showed higher expression in EBV-positive NPC, suggesting that EBV infection may modulate the autophagy machinery. ATG3 and ATG4C protein expression was also significantly more frequent in EBV-positive tumors. Furthermore, elevated ATG4B and ATG4D expression was associated with reduced distant metastasis, whereas reduced ATG4D mRNA levels were associated with increased metastatic rates and shorter distant metastasis-free survival. Although validation in larger cohorts is required, these findings support the potential utility of autophagy-related molecules as biomarkers for disease stratification and clinical outcomes and suggest that virus-associated regulation of autophagy may contribute to NPC biology.

Overall, this collection underscores autophagy as both a fundamental regulator of tumor biology and a potential therapeutic vulnerability. Rather than viewing autophagy simply as a pathway to inhibit or activate, future studies should define its specific form, function, cellular compartment, and disease context. Combining autophagy-directed strategies with targeted therapies, chemotherapy, cellular stress-inducing agents, or interventions targeting apoptosis and mitochondrial function may provide opportunities to overcome therapeutic resistance. Rigorous biomarker development and carefully designed clinical trials will be essential to identify patients and treatment settings most likely to benefit. Collectively, the studies in this collection advance a broader understanding of autophagy as a central component of tumor adaptation, inflammation, and therapeutic response.

